# Oxalate Content of Taro Leaves Grown in Central Vietnam

**DOI:** 10.3390/foods6010002

**Published:** 2017-01-01

**Authors:** Hang Du Thanh, Hai Phan Vu, Hai Vu Van, Ngoan Le Duc, Tuan Le Minh, Geoffrey Savage

**Affiliations:** 1 Faculty of Animal Husbandry and Veterinary Medicine, Hue University of Agriculture and Forestry, Hue city, Vietnam; hai.phanvu@huaf.edu.vn (H.P.V.); hai.vuvan@gmail.com (H.V.V.); le.ngoan@huaf.edu.vn (N.L.D.); 2 Faculty of Hospitality & Tourism, Hue University, Hue city, Vietnam; tuanle1709@gmail.com; 3 Food Group, Department of Wine, Food and Molecular Biosciences, Faculty of Agriculture and Life Sciences, Lincoln University, Canterbury 7647, New Zealand; Savage@lincoln.ac.nz

**Keywords:** total, soluble and insoluble oxalates, taro leaves, calcium, calcium availability

## Abstract

Leaves were harvested from four different cultivars of *Colocasia esculenta* and three cultivars of *Alocasia odora* that were growing on nine different farms in central Vietnam. The total, soluble and insoluble oxalate contents of the leaves were extracted and measured using HPLC chromatography. Total calcium determinations were also carried out on the same samples. The total oxalate content of the leaves ranged from 433.8 to 856.1 mg/100 g wet matter (WM) while the soluble oxalate ranged from 147.8 to 339.7 mg/100 g WM. The proportion of soluble oxalate ranged from 28% to 41% (overall mean 35%) of the total oxalate content of the leaves. The equivalent insoluble oxalate proportion ranged from 59% to 72% of the total (overall mean 65%). There was little difference between the *Colocasia esculenta* and *Alocasia odora* taro cultivars, although the total oxalate content was significantly higher in *Alocasia odora* cultivars. The overall mean total calcium content was 279.5 mg/100 WM and the percentage of insoluble calcium bound as calcium oxalate ranged from 31.7% to 57.3% of the total calcium content (overall mean 47.1%). The oxalate content in taro leaves is a major factor to consider when different cultivars of taro are recommended for human or animal consumption.

## 1. Introduction

Taro (*Colocasia esculenta)* has been reported to yield up to 370 t/ha/year of foliage (leaves and petioles) [1]. In central Vietnam, there are seven main varieties of taro, which are widely grown and have local names. The characteristics of these are shown in Table 1. The estimated production of taro foliage is approximately 200 t/ha, with the leaves representing some 50% of the foliage dry matter. Taro leaves are rich in protein (3.2% to 3.9% of wet matter (WM)) while the petioles are rich in soluble carbohydrates [1,2,3,4]. Taro is grown extensively as tubers for human consumption while the foliage (leaves and petioles) is used as a feed ingredient, mainly for pigs [1,3,4]. Taro foliage is rich in nutrients, such as crude protein and calcium, and would therefore provide a useful vegetable for human use [4]. Taro leaves are often consumed as a vegetable in central Vietnam but their consumption is limited by the sharp taste of certain cultivars.

The occurrence of fine needle-like crystals or raphides, made up of calcium oxalate, in tropical root crops and particularly in aroids such as taro (*Colocasia*), taro (*Xanthosoma*), giant taro, giant swamp taro and elephant foot yam have been considered either the cause or the contributing cause to the acridity of the tubers of these root crops [5]. Holloway et al. [5] were the first to report the total and insoluble oxalate content of the leaves of nine different cultivars of taro grown in Fiji. The authors noted that not all of the leaves were considered edible by Fijians. The total oxalate of the nine cultivars ranged from 278 to 574 mg/100 g wet matter (WM), with the edible leaves generally having lower levels of total oxalates than the leaves considered inedible. In this study, insoluble oxalate was not observed in all cultivars. More recently, it was shown that the oxalate content of young taro leaves grown in green houses in New Zealand contained high levels of total, soluble and insoluble oxalate (respectively 589, 442 and 147 mg oxalate/100 g WM) [6]. The cultivar of these taro plants, which did not form tubers, was not known, but the original plants were imported from Tonga. In 2011, Hang et al. [7] showed that the total oxalate of the leaves of eight different cultivars of taro grown in central Vietnam ranged from 115 to 362 mg/100 g WM while the soluble oxalate contents were more variable (4.7 to 80 mg/100 g WM).

In 2013, Hang and Binh [8] showed that the leaves of Mon Tim (*Alocasia odora*) contained high levels of oxalates in the leaves (total, soluble and insoluble oxalate of 661, 345 and 316 mg/100 g WM, respectively) which were much higher than the levels found in Mon Nuoc and Chia Voi (*Colocacia esculenta*) (mean values of 378, 157 and 221 mg/100 g WM, respectively, for total, soluble and insoluble oxalates).

Different cultivars of taro are grown in various parts of central Vietnam. Chum grows well in sandy and clay soils and produces its best corms in high humus-containing soils. It is a cultivar that is appreciated for its good taste and smell. Cultivars Chum, Ngot and So Trang are preferred by people because they grow well and have a low incidence of itchiness when handled and eaten. The corms of So Trang are appreciated for their aromatic, buttery taste and soft texture when cooked.

Therefore, the main limitation of the use of taro leaves as a vegetable for humans is the presence of oxalates which can form non-absorbable salts with Ca, Fe and Mg, rendering these minerals unavailable [9] and increasing the risk of kidney stone formation when excess oxalates are excreted by the kidneys [10]. Holloway et al. [5] were the first to observe that a large proportion of the total calcium of taro leaves would be locked up in taro leaves as calcium oxalate, leaving very small amounts of free calcium in the leaf tissue. In contrast, only 10% of the total calcium content of young New Zealand–grown taro leaves was locked up as insoluble calcium oxalate [6].

Therefore, this study was conducted to determine the oxalate contents of the fresh leaves of several varieties of taro commonly grown in central Vietnam to evaluate their use as a cheap food for human or animal use. In addition, the total calcium content was measured in the same samples to determine how much of the total calcium content was unavailable in the leaf tissue.

## 2. Materials and Methods

### 2.1. Harvesting and Processing

The taro plants used in this experiment were grown on nine different farms with similar sandy soils located close to the experimental farm. The plants were not fertilized during the growing season and they were irrigated to maintain steady growth throughout the experimental period. A total of five kg of young growing leaves were harvested from five representative plants from each cultivar plot (So Trang, Tron, Tia, Chum, Quang, Huong and Sap Vang). These samples were mixed together and chopped into 10–20 mm pieces. The chopped pieces were then mixed for an additional 20 min. Three representative subsamples were sampled and dried at 65 °C for 18 h. Three hundred g of dried material from each variety were then sealed in plastic bags and stored until analysis commenced. Each sample was ground to a fine powder using a Sunbeam Multi-Grinder (Model No. EMO 400, Sunbeam Corporation Limited, Botany, NSW, Australia) and the residual moisture was determined in triplicate [11] by drying to a constant weight in an oven at 105 °C for 24 h.

### 2.2. Oxalate Determination

The total and soluble oxalate contents of the individual finely ground samples (~0.5 g) were determined using the method outlined by Savage et al. [12]. Each sample was extracted to measure the total oxalate content and three replicates were extracted to measure the soluble oxalate contents. Forty mL of 0.2 M HCL (Aristar, BDH Chemicals, Ltd., Poole, Dorset, UK) was added to the flasks for the total oxalate extraction and 40 mL of high purity water was added for the extraction of soluble oxalates. All flasks were placed in an 80 °C shaking water bath for 20 min. The solutions were allowed to cool to 20 °C and then made up to 100 mL with 0.2 M HCL for total oxalate, and high purity water, for soluble oxalate, respectively. The extracts in the volumetric flasks were filtered through a cellulose acetate syringe filter with a pore size of 0.45 µm (dismic-25cs, Advantec, Dublin, CA, USA) into 1 mL glass HPLC vials. The samples were analyzed with a high performance liquid chromatography (HPLC) system, using a 300 mm × 7.8 mm Rezex ion exclusion column (Phenomenex Inc., Torrance, CA, USA) attached to a Cation-H guard column (Bio-Rad, Richmond, CA, USA) held at 25 °C. The analysis was performed by injecting 20 µL of sample or standard onto the column using an aqueous solution of 25 mmoL sulphuric acid (HPLC grade Baker Chemicals, Phillipsburg, NJ, USA) as the mobile phase, then pumped isocratically at 0.6 mL/min, with peaks detected at 210 nm. The HPLC equipment consisted of a Shimadzu LC-10AD pump, CTO-10A column oven, SPD-10Avp UV-Vis detector (Shimadzu, Kyoto, Japan) and a Waters 717 plus auto-sampler (Waters, Milford, MA, USA). Data acquisition and processing was undertaken using the Peak Simple Chromatography Data System (Model 203) and Peak Simple software version 4.37 (SRI Instruments, Torrance, CA, USA). The oxalic acid peak was identified by comparing the retention time with a standard solution and by spiking an already-filtered sample containing a known amount of oxalic acid standard. The insoluble oxalate content was calculated by the difference between the total and soluble oxalate contents [5]. The final oxalate values of all samples were converted to mg/100 g WM of the original material, taking into account the moisture content of each sample.

### 2.3. Calcium Determination

Total calcium content was analyzed using an atomic absorption spectrometer (AOAC, method 945.46) [13]. Calibration of the measurements was performed using commercial standards using AOAC method 991.25 [13]. The calcium bound up in insoluble oxalate was calculated assuming that insoluble oxalate was predominantly calcium oxalate and that calcium was 31.28% of this molecule.

### 2.4. Statistical Analysis

All analyses were carried out in triplicate and the results are presented as mean values ± standard error. Statistical analysis was performed using one-way analysis of variance (Minitab version 16, Minitab Ltd., Brandon Court, Progress way, Coventry, UK).

## 3. Results

In this study, seven cultivars derived from two taro species, *Colocasia esculenta* and *Alocasia odora*, were evaluated for their potential use for humans and animals. The levels of oxalate and the amount of calcium bound up in the insoluble oxalate fraction will have an important effect on their efficient utilization within the body. Table 2 shows the individual values of the total, soluble and insoluble oxalate in each cultivar and the mean values for each group. Total oxalate was the lowest in Chum and the highest in Quang cultivars (433.8 vs. 856.1 mg/100 g WM). Soluble oxalate ranged widely between the different taro cultivars (147.8 to 339.7 mg/100 g WM). Overall, the proportion of soluble oxalate varied within the cultivars from 27.8% to 40.7% of the total oxalate.

Statistical analysis showed that there were significant differences within the two groups of cultivars (*P* < 0.05) for all the parameters measured. However, analysis of the mean comparisons between the two cultivar groups showed that only the *Alocasia oderata* cultivars contained significantly higher levels of total oxalates *(P* < 0.05) when compared to the *Colocasia esculenta* cultivars (635.2 vs. 668.8 mg total oxalate/100 g WM). All the other mean values for these two cultivars were similar.

Table 3 shows the values for the total calcium and the calcium bound up in the insoluble oxalate fraction in the taro cultivars evaluated.

The total calcium content of the fresh leaves ranged from 239.5 to 309.4 (mean 279.5) mg calcium/100 g WM, while the ratio of insoluble calcium to total calcium in the fresh leaves ranged from 31.7% to 57.4% (Table 3). Cultivars Quang and Tron contained the highest total calcium content of the taro cultivars evaluated and they also contained the highest levels of calcium bound in the insoluble oxalate fraction. There were no significant differences in the mean values of any of the other parameters measured for the two groups of cultivars evaluated.

## 4. Discussion

Earlier publications have suggested that the concentrations of oxalate in plants are influenced by environmental and biological factors, fertilizer application, light intensity, plant variety and cultivar [14,15]. In this experiment the environmental and growth factors were the same for all of the cultivars evaluated. This experiment showed that significant (*P* < 0.05) differences between the different cultivars could be observed. The observed values of the total oxalate of the leaves in this experiment were similar to the values reported for taro grown in Fiji [5] and also those reported by Noonan and Savage [16]. The total oxalate content of taro leaves grown in New Zealand [6] was lower than that observed for plants grown in Vietnam in this experiment. However, they showed that the total oxalate content of the young taro leaves was higher (589 mg/100 g WM) than in older leaves (433 mg/100 WM). In contrast, the total mean calcium content of New Zealand–grown leaves was 169 mg calcium/100 g WM with the insoluble calcium content ranging from 5.8% to 10%. Later studies showed that both Maori and Japanese cultivars of taro contained significant amounts of oxalates but the soluble oxalate content of these could be reduced significantly by feeding with calcium-containing foods [17].

Soluble oxalate in food items is more bioavailable than insoluble oxalate [18]. Soluble oxalate can bind to calcium from other food sources in the intestine or can be absorbed and increase the risk of kidney stone formation [18,19]. Further studies should focus on the effect on oxalate concentrations of processing and cooking prior to consumption as taro leaves are not eaten raw; they are commonly boiled for 10 min to reduce the sharp taste by leaching out soluble oxalates. In addition, cooked taro leaves are rarely eaten alone; they are commonly eaten with a fish sauce or cooked with fish which would add soluble calcium to the food mix, which then could reduce the soluble oxalate content.

Overall, the total oxalate content of the leaves of the seven different cultivars of taro grown in Vietnam in this study varied from 433.8 to 856.1 mg/100 g WM, while the soluble oxalate content as a proportion of the total oxalate content ranged from 27.8% to 40.7% with a mean of 33.4%. While there was little difference between the mean values of the oxalate content of the Alocacia and Colocacia species, overall there was a greater difference within the cultivars. This could have implications on the choice of which cultivars should be grown for human and for animal consumption.

## 5. Conclusions

The total oxalate contents of taro leaves of the cultivars investigated ranged from 433.8 to 856.1 mg/100 g WM, while the soluble oxalate contents ranged from 27.8% to 40.7% of the total oxalate contents. Overall, the data showed that between 31.7% and 57.4% of the total calcium content of the leaves was bound in an insoluble form; this suggests that the total oxalate and the bound calcium contents are major factors to consider when different taro cultivars are recommended for human consumption.

## Figures and Tables

**Table 1:** Vietnamese and scientific names and key characteristics of common taro cultivars grown in central Vietnam.

Vietnamese Name	Key Characteristics	Stem Colours	Leaf Colours
*Colocasia esculenta* cultivars			
So Trang	One main corm in the center surrounded by many daughter cormels	Green	Light green with a purple mark between the leaf and the stem
Tron	One main big corm and little cormels	Dark green	Rough, light green
Tia	One main corm in the center surrounded by some purple cormels	Purple	Light green with prominent veins
Chum	One main corm in the center surrounded by many cormels	Light green	Dark green
*Alocasia odora* cultivars			
Quang	One main big cylindrical corm surrounded by some cormels	Purple	Light green with a purple mark between the leaf and the stem, one dark spot in the middle of the leaf
Huong	One main big spotty purple fleshed cylindrical corm surrounded by some cormels	Light purple	Rough green with prominent veins
Sap Vang	One main big cylindrical yellow fleshed corm surrounded by some little cormels	Green	Large, dark green

**Table 2:** Total, soluble and insoluble oxalate content (mg/100 g WM) in the leaves of seven different taro cultivars grown in central Vietnam (mean values ± SE).

Taro Cultivar	Dry Matter (%)	Total Oxalate (mg/100 g WM)	Soluble Oxalate (mg/100 g WM)	Insoluble Oxalate (mg/100 g WM)	Proportion of Soluble/Total (%)
*Colocasia esculenta* cultivars					
So Trang	18.33 ± 0.07	531.3 ± 9.2 ^c^	147.8 ± 4.5 ^d^	383.5 ± 6.2 ^b^	27.8 ± 0.5 ^c^
Tron	19.33 ± 0.06	835.4 ± 7.7 ^a^	339.7± 7.3 ^a^	495.7 ± 9.9 ^a^	40.7 ± 0.9 ^a^
Tia	19.31 ± 0.22	740.3 ± 5.9 ^b^	253.3 ± 4.7 ^b^	487.0 ± 10.3 ^a^	34.2 ± 0.9 ^b^
Chum	18.81 ± 0.02	433.8 ± 7.9 ^d^	170.8 ± 3.2 ^c^	263.0 ± 5.3 ^c^	39.4 ± 0.4 ^a^
*Alocasia odora* cultivars					
Quang	18.31 ± 0.04	856.1 ± 10.0 ^a^	288.8 ± 7.7 ^a^	567.2 ± 8.1 ^a^	33.7 ± 0.7 ^a^
Huong	19.19 ± 0.04	631.0 ± 4.8 ^b^	227.2 ± 3.8 ^b^	403.8 ± 6.0 ^b^	36.0 ± 0.7 ^a^
Sap Vang	18.76 ± 0.06	519.3 ± 6.3 ^c^	157.8 ± 3.7 ^c^	361.4 ± 6.39 ^c^	30.4 ± 1.0 ^b^
Mean values					
*Colocasia esculenta* cultivars	18.87 ± 0.03	635.2 ± 92.4 ^a^	227.9 ± 43.6	407.3 ± 54.4	35.5 ± 2.9
*Alocasia odora* cultivars	18.77 ± 0.03	668.8 ± 99.0 ^b^	224.6 ± 37.8	444.1 ± 54.3	33.4 ± 1.6
Overall mean	18.83 ± 0.16	649.6 ± 62.4	226.5 ± 27.4	423.1 ± 38.3	34.6 ± 1.7

^a,b,c,d^ Means with different letters within columns (compared within cultivars) differ at *P* < 0.05.

**Table 3:** Mean values (±SE) for total calcium content (mg/100 g WM) and calcium in insoluble oxalate in the leaves of seven different taro cultivars grown in central Vietnam.

Taro Cultivars	Total Calcium (mg/100 g WM)	Calcium in Insoluble Oxalate (mg/100 g WM)	Insoluble Calcium/Total Calcium (%)
*Colocasia esculenta* cultivars			
So Trang	239.5 ± 2.2 ^d^	119.9 ± 1.9 ^b^	50.1 ± 0.8 ^a^
Trom	305.4 ± 4.3 ^a^	155.1 ± 3.1 ^a^	50.8 ± 1.7 ^a^
Tia	283.8 ± 1.5 ^b^	152.3 ± 3.2 ^a^	53.7 ± 1.2 ^a^
Chum	259.5 ± 3.0 ^c^	82.2 ± 1.6 ^c^	31.7 ± 0.6 ^b^
*Alocasia odora* cultivars			
Quang	309.4 ± 3.5 ^a^	177.4 ± 2.5 ^a^	57.4 ± 1.4 ^a^
Huong	277.6 ± 2.6 ^b^	126.3 ± 1.9 ^b^	45.5 ± 1.1 ^b^
Sap Vang	281.0 ± 5.2 ^b^	113.1 ± 1.2 ^c^	40.3 ± 0.4 ^c^
Mean values			
*Colocasia esculenta* cultivars	272.1 ± 14.3	127.4 ± 17.0	46.6 ± 5.0
*Alocasia odora* cultivars	289.3 ± 10.1	138.9 ± 19.6	47.7 ± 5.1
Overall mean	279.3 ± 9.2	132.4 ± 12.0	48.4 ± 3.1

^a,b,c,d^ Means with different letters within columns (compared within cultivars) differ at *P* < 0.05.
